# Acceptability of virtual reality to screen for dementia in older adults

**DOI:** 10.1186/s12877-024-05115-w

**Published:** 2024-06-05

**Authors:** Joyce Siette, Patrick J. Adam, Celia B. Harris

**Affiliations:** The MARCS Institute for Brain, Behaviour and Development, Western Sydney University, Westmead, NSW 2145 Australia

**Keywords:** Virtual reality, Older adults, Acceptability, Cognition digital health

## Abstract

**Background:**

Early detection of dementia and cognitive decline is crucial for effective interventions and overall wellbeing. Although virtual reality (VR) tools offer potential advantages to traditional dementia screening tools, there is a lack of knowledge regarding older adults’ acceptance of VR tools, as well as the predictors and features influencing their adoption. This study aims to (i) explore older adults’ perceptions of the acceptability and usefulness of VR diagnostic tools for dementia, and (ii) identify demographic predictors of adoption and features of VR applications that contribute to future adoption among older adults.

**Methods:**

A cross-sectional study was conducted involving community-dwelling older adults who completed online questionnaires covering demographics, medical history, technology acceptance, previous usage, and perceived usefulness and barriers to VR adoption. Multiple linear regression was employed to assess relationships between sociodemographic factors, prior technology use, perceived ease, usefulness, and intention to adopt VR-based diagnostic tools.

**Results:**

Older adults (*N* = 77, *M*_age_ = 73.74, *SD* = 6.4) were predominantly female and born in English-speaking countries. Perceived usefulness of VR applications and educational attainment emerged as significant predictors of the likelihood to use VR applications for dementia screening. Generally, older adults showed acceptance of VR applications for healthcare and dementia screening. Fully immersive applications were preferred, and older adults were mostly willing to share electronic information from screening with their healthcare providers.

**Conclusions:**

The field of research on VR applications in healthcare is expanding. Understanding the demographic characteristics of populations that stand to benefit from healthcare innovations is critical for promoting adoption of digital health technologies and mitigating its barriers to access.

**Supplementary Information:**

The online version contains supplementary material available at 10.1186/s12877-024-05115-w.

## Introduction

The demographic landscape is undergoing a significant transformation, with the World Health Organization [1] projecting that the global population aged 65 and above will rise to 2.1 billion by 2050. Alongside this demographic shift, there is a parallel increase in the prevalence of dementia [2], necessitating innovative approaches in healthcare to alleviate the associated disease burden [3]. The utilisation of virtual reality (VR) applications has emerged as a novel and non-invasive method for assessing physical and cognitive functioning in older individuals, offering a promising avenue for addressing the challenges posed by the expanding ageing population [4].

Virtual reality, characterised by its immersive three-dimensional environments, has found applications across diverse industries, including education, entertainment, and healthcare [5–8]. Within the healthcare domain, all forms of VR technology (semi-immersive, non-immersive and fully immersive) have been shown to be particularly valuable for cognitive and physical assessments, as well as interventions and rehabilitation [9]. For older adults, VR applications also provide a non-intrusive means of evaluating cognitive and physical capabilities, demonstrating superiority over conventional approaches [10]. For example, VR simulations can create immersive environments that mimic real-world scenarios, allowing healthcare professionals to assess cognitive functions such as spatial memory and problem-solving skills in a controlled and interactive setting [11, 12]. Similarly, VR-based exercises and therapies can be tailored to target specific physical abilities like balance, mobility, and coordination, offering a more engaging and effective rehabilitation experience for older adults compared to traditional methods [13].

Recent studies have further reported positive outcomes from the use of VR applications with older adults, showcasing its potential across various healthcare domains [14–16]. Orr et al. [15] demonstrated that VR interventions positively influenced cognitive health, physical wellbeing, and emotional resilience among older adults, with participants engaging with VR reported improvements in memory and attention. The immersive and interactive nature of VR experiences also facilitated physical activities, contributing to improved mobility and overall physical health. Additionally, participants reported increased engagement, reduced feelings of isolation, and heightened emotional resilience, emphasising the psychosocial benefits of VR technology for older adults. The decision to prioritise VR for dementia screening is based on its ability to replicate cognitive challenges specific to dementia, such as memory tasks and spatial navigation, offering critical insights for clinicians and older adults in a non-stressful setting [11, 12].

Despite the promise, the adoption of VR applications for healthcare, particularly in the context of dementia screening among older adults, faces a multitude of barriers and facilitators. The Technology Acceptance Model (TAM) serves as a guiding framework, suggesting that the intention to use novel technologies hinges on perceived ease of use and usefulness [17]. While the TAM has been extensively employed to assess VR acceptance, it is essential to acknowledge that older adults with sensory impairments may encounter challenges in fully engaging with VR technologies. Both Shu and Woo [18] and Facal et al. [10] highlighted the impact of sensory impairments on the acceptance and usability of VR applications among older adults. Factors such as cybersickness and medication-induced nausea further complicate the interaction with VR applications, especially within an older demographic [19]. These studies highlight the importance of considering the unique challenges faced by older individuals including those with sensory impairments and other medical complications when evaluating the feasibility and acceptance of VR technology in healthcare settings.

Beyond individual differences in suitability based on health status, the adoption of innovative technologies is deeply intertwined with sociodemographic factors, health status, and technological experience. These factors often shape the acceptability and usability of novel treatments or technologies in healthcare, such as predicting acceptance of the COVID-19 vaccine among different groups in the United States [20]. Similarly, research on technology usage among older adults has highlighted the influence of sociodemographic factors in determining preferences and adoption [21, 22].

Despite the wealth of research demonstrating the potential benefits of VR applications in the screening and diagnosis of dementia, there exists a notable gap in understanding how sociodemographic factors, health status, technological acceptance and experience variables collectively influence older adults’ likelihood to use VR for dementia screening. There is thus a clear need to identify predictors of adopting VR applications for screening purposes, with the goal of recognising early adopters and developing targeted interventions. Additionally, comprehending these predictors will enhance our understanding of which populations are less inclined to embrace novel dementia screening tools. This insight will enable a more targeted approach to the integration of VR technologies in healthcare, ensuring the right approach for the right person as well as informing strategies to increase acceptability.

This study thus aimed to (i) explore older adults’ acceptance, preferences, and likelihood of adopting VR applications (fully immersive, semi-immersive and non-immersive) for cognitive screening, and (ii) understand how sociodemographic factors, health status, and perceptions of VR applications contribute to older adults’ likelihood of adopting VR applications for cognitive screening. It was hypothesised that older adults will demonstrate varying levels of acceptance, preferences, and likelihood of adopting VR applications for cognitive screening. Factors such as familiarity with technology, previous VR experience, and perceived benefits are expected to influence their attitudes and intentions towards using VR.

## Methods

### Design

An online cross-sectional survey was distributed between April and October 2022. This study was approved by the Western Sydney University Human Research Ethics Committee (H14896).

### Eligibility criteria

Participants needed to be aged 65 years or above, possess a sufficient level of English proficiency to effectively comprehend and communicate in English during the research study, and be capable of giving consent to be part of the study. Additionally, participants needed to reside in Australia to be eligible for participation. Exclusion criteria further specified that individuals with a diagnosis of dementia were not eligible to participate.

### Recruitment

A diverse pool of participants was recruited through multiple channels, ranging from traditional methods such as flyer drops and local print advertisements to leveraging the expansive reach of social media. The recruitment strategy also benefited from networks within the research team and was disseminated through e-newsletters.

### Measurement

The survey, administered via Qualtrics, consisted of five sections: demographics, health status, current technology usage, appraisal and acceptance of VR, and VR application preferences. It comprised 36 questions and took around 30 min to complete.

Demographics, health status and current technology use questions in this study were adopted from previous national survey studies conducted with older Australians [23–25]. The latter questions on appraisal and acceptance of VR technology were further adapted from the Technology Acceptance Model (TAM) [17] to assess participants’ perceptions of technology acceptance and usability according to our study’s objectives. Participants reviewed the participant information sheet, and consent was initially obtained electronically through e-signatures and checkboxes on Qualtrics. Participants had the option to exit the survey at any point (refer to the Supplementary Material for a copy of the survey).

i) ***Demographics***: Following previous national survey studies [23–25], nine questions regarding participants’ age, gender, country of birth, marital and employment status, and postcode. Educational attainment was split into low (minimal or no formal education), middle (intermediate levels of education such as secondary education or vocational training) and high attainment (completed tertiary education). Postcode data in our study served a dual purpose: firstly, to distinguish participants based on geographical locality (urban or regional), enabling exploration of potential regional variations in responses. Secondly, postcodes were used to index participants’ socioeconomic status (SES) through the Socio-Economic Indexes for Areas (SEIFA).
ii) ***Health Status***: Participants were asked to disclose if they had been diagnosed by a healthcare professional with any of the following health conditions: chronic heart disease, diabetes, stroke, hearing and sight impairments, hypertension, asthma, depression and anxiety.
iii) ***Current Technology Usage***: Six questions were asked on type and frequency of technology use for healthcare purposes. These questions were based on a prior national study exploring Australian older adults’ use of technology [23]. Participants were asked if they used their phone (landline or mobile), tablets (e.g., iPad), computer, wearables (e.g., FitBit) and smart home devices (e.g., Google Nest) for healthcare purposes. They were also asked how frequently they used their devices for healthcare reasons: *a few times a week, once a day, a few times a week, once a week*, or *once a month*. Participants were further asked about the type and frequency of use of various applications for health purposes (e.g., *games*, *social media*, *web browsers*, *e-health)*.
iv) ***Appraisal and Acceptance of VR***: Participants were first provided with a definition of VR (“Virtual reality can be defined as a simulated environment in which an user experiences telepresence”) before being asked about their prior experiences with VR applications (e.g., fully immersive, non-immersive, semi-immersive and augmented reality, with each of these options being provided with a definition and example; see Supplementary Material), perceptions on ease of use and usefulness, and acceptance and appraisal of VR usage for healthcare and dementia screening. Participants were asked to rate VR applications on usefulness, ease to learn and use, acceptability and usefulness for older individuals on a five-point Likert scale ranging from *strongly disagree* to *strongly agree*. Prior experiences with VR sickness were also collected. Participants were also asked if they would personally recommend VR applications for dementia screening and were asked about how likely they would be to personally use VR applications.
v) ***VR Applications Preferences***: Ratings on a five-point Likert scale supported participants’ perspectives on various aspects of VR applications for memory assessments, including usefulness, ease of learning and use, and acceptability.

### Statistical analysis

Descriptive and frequency statistics were initially conducted to summarise the sociodemographic, health status, current technology use, VR appraisal, acceptance, prior usage, and preference variables and examine overall levels of acceptance and likelihood to adopt. For descriptive statistics, means and standard deviations were calculated for continuous variables, while frequencies and percentages were computed for categorical variables.

To explore the predictors of participants’ likelihood to use VR applications for dementia screening, a standard multiple regression analysis was performed. Sociodemographic, health status, and VR acceptance variables were included as independent variables, while the dependent variable was the participants’ likelihood to use VR applications. Multiple regression was deemed appropriate as it allows for the examination of the unique contribution of each predictor variable while controlling for the others [26].

Assumptions of multiple regression were checked to ensure the validity of the analysis and prior to interpretation of the results. Firstly, the linearity assumption was assessed by examining scatterplots of the independent variables against the residuals. This visual inspection compared standardized residual values to standardized predicted values. It was observed that the standardized residuals were evenly distributed across the range of predictor values, and no nonlinear pattern was detected, supporting the assumption of linearity [27]. Secondly, multicollinearity was assessed using variance inflation factors (VIFs), with all VIF values within an acceptable range (VIF < 10), indicating no multicollinearity issues [27]. Thirdly, homoscedasticity was examined through residuals plots, and no systematic patterns were identified, confirming homoscedasticity [26].

Normality of residuals was assessed through histograms, Q-Q plots, and the Shapiro-Wilk test. While visual inspection indicated approximately normal distribution, the Shapiro-Wilk test confirmed non-significance (*p* > .05), suggesting that the assumption of normality was met [26]. The Durbin-Watson statistic was calculated to assess the independence of residuals, with the value falling within an acceptable range (1.5 < DW < 2.5), confirming independence [27]. IBM SPSS Statistics (version 27) was used for all statistical analyses. The significance level for all tests was set at *p* < .05.

## Results

Out of the initial 101 clicks on the survey link, 78 respondents proceeded to provide consent, while one individual was deemed ineligible. Subsequently, 77 respondents completed the entire survey. The average time taken by respondents to complete the survey was 12.4 min (SD = 9.2). The sample’s sociodemographic characteristics are summarised in Table 1. The majority of participants belonged to the 65–74 age group, with an average age of 73.7 years (*SD* = 6.4). Female-identifying participants constituted 64.9% of the sample, with the remainder identifying as male. A significant portion of participants were born in English-speaking countries, belonged to the highest socioeconomic quintile, were either retired or unemployed, and lived in major cities. Furthermore, a majority of the sample exhibited middle to high levels of educational attainment. Health status of the sample can be seen in Table 2. Hypertension was the most commonly reported health condition (35.1%) while stroke was the least common (3.9%). Almost three-quarters (70.2%) had either one or two chronic conditions.

**Table 1:** Summary of participants’ sociodemographic characteristics (*N* = 77)

Sample Characteristic	*n*	%	Mean (SD)
**Age**			73.7 (6.4)
65–74	44	57.1	
75–84	27	35.1	
85+	6	7.8	
**Gender**			
Female	50	64.9	
Male	26	33.8	
Non-binary	1	1.3	
**Place of birth**			
English-speaking	51	66.2	
Non-English speaking	26	33.8	
**Socioeconomic status**			
1st quintile (lowest)	22	28.6	
2nd quintile	4	5.2	
3rd quintile	12	15.6	
4th quintile	6	7.8	
5th quintile (highest)	33	42.9	
**Locality**			
Major city	64	83.1	
Inner/Outer Regional	13	16.9	
**Educational attainment**			
Low	8	10.4	
Middle	40	51.9	
High	29	37.7	
**Marital status**			
Single/Widowed/Divorced	31	40.3	
Married/De facto	44	57.1	
No response	2	2.6	
**Employment status**			
Employed/Student	7	9.1	
Retired/Unemployed	68	88.3	
No response	2	2.6	

**Table 2:** Participants’ health characteristics

Health Characteristic	*n*	%	Mean (SD)
**Diagnosed condition**			
Chronic heart disease	7	9.1	
Diabetes	19	24.7	
Stroke	3	3.9	
Sight impairment	10	13	
Hearing impairment	7	9.1	
Hypertension	27	35.1	
Asthma	16	20.8	
Depression/Anxiety	22	28.6	
**Total number of chronic conditions**			1.7 (1.1)
None	11	14.3	
One	25	32.5	
Two	29	37.7	
Three	6	7.8	
Four	4	5.2	
Five	2	2.6	

Over half of the sample (*n* = 43, 53.3%) were very interested in using technology to improve their health. Mobile phones were the most commonly used device for general purposes, followed by tablets and computers, landline phones, wearables and smart home devices (Table 3). Frequency of usage varied between the device types. Over half of the sample used either mobile or landline phones multiple times per day. One-fifth of the sample used social media, web browsers or pre-installed applications multiple times per day. Streaming services and games were less commonly used.

**Table 3:** Frequency and type of technology and application usage (*N* = 77)

	*n* (%)				
	Few times a day	Daily	Few times a week	Weekly	Monthly
**Technology type**					
Landline	17 (22.1)	4 (5.2)	1 (1.3)	15 (19.5)	15 (19.5)
Mobile	31 (40.3)	5 (6.5)	1 (1.3)	11 (14.3)	20 (26)
Tablet	14 (18.2)	3 (3.9)	6 (7.8)	12 (15.6)	18 (23.4)
Computer	14 (18.2)	1 (1.3)	7 (9.1)	11 (14.3)	20 (26)
Wearables	13 (16.9)	2 (2.6)	0 (0)	2 (2.6)	3 (3.9)
Smart home	9 (11.7)	3 (3.9)	0 (0)	5 (6.5)	3 (3.9)
**Application type**					
Games	12 (15.6)	2 (2.6)	1 (1.3)	3 (3.9)	5 (6.5)
Social media	18 (23.4)	3 (3.9)	8 (10.4)	11 (14.3)	18 (23.4)
Web browsers	20 (26)	1 (1.3)	8 (10.4)	7 (9.1)	20 (26)
Native	16 (20.8)	1 (1.3)	12 (15.6)	13 (16.9)	14 (18.2)
Streaming	8 (10.4)	5 (6.5)	7 (9.1)	11 (14.3)	12 (15.6)
E-health	12 (15.6)	3 (3.9)	7 (9.1)	12 (15.6)	9 (11.7)

Note. Native applications refer to applications which come preinstalled on devices (e.g. cameras, calculators)

Approximately 41.6% of participants had prior experience with VR or AR games, with fully immersive games being the most common (*n* = 22, 28.6%), followed by non-immersive (*n* = 11, 14.3%), semi-immersive (*n* = 10, 13%), and AR (*n* = 5, 6.5%). Table 4 illustrates participants’ appraisals and perceptions of VR applications. About 35.1% of the sample found VR applications useful, and over half either agreed or strongly agreed that these applications would be easy to learn, acceptable, and useful for older individuals. Additionally, more than three-quarters of the sample expressed a likelihood to use VR applications for dementia screening. Only 11.7% reported experiencing VR sickness. The majority (*n* = 40, 51.9%) were very likely, while 26% (*n* = 20) were likely to personally use VR applications for dementia screening. A small proportion reported being unlikely (*n* = 4, 5.2%), very unlikely (*n* = 1, 1.3%), or responded neutrally (*n* = 2; 2.6%).

**Table 4:** Appraisal and acceptance of VR technologies

	*n* (%)				
Question	Strongly disagree	Disagree	Neutral	Agree	Strongly agree
Useful	2 (2.6)	2 (2.6)	9 (11.7)	22 (28.6)	27 (35.1)
Easy to learn	6 (7.8)	4 (5.2)	16 (20.8)	28 (36.4)	14 (18.2)
Easy to use	6 (7.8)	2 (2.6)	28 (36.4)	26 (33.8)	6 (7.8)
Acceptable for older adults	2 (2.6)	2 (2.6)	16 (20.8)	37 (48.1)	10 (13)
Useful for older adults	2 (2.6)	0 (0)	16 (20.8)	35 (45.5)	13 (16.9)
Personally recommend	4 (5.2)	2 (2.6)	15 (19.5)	29 (37.7)	16 (20.8)

Note. Not all participants responded to each question. Percentages are representative of the entire sample

Participants’ preferences for features of VR applications were noted, with the majority (*n* = 46, 59.7%) expressing a preference for online websites or downloadable programs for computers, tablets, or phones. Fully immersive applications were the top choice (45.5%), followed by semi-immersive (20.8%) and non-immersive (2.6%). Table 5 details potential VR technology descriptions and participants’ reported satisfaction levels. In terms of frequency of usage, about 24.7% of the sample indicated a willingness to engage weekly in a hypothetical VR application assessing cognitive abilities. Regarding information sharing, most participants (*n* = 50) preferred sharing with their general practitioner, followed by family members (28.6%), partners (14.3%), and friends (2.6%). Only five (6.5%) participants opted not to share the information with others.

**Table 5:** Proportion of satisfaction ratings with current VR technologies assessing cognitive ability

	*n* (%)				
Description of VR technology	Very unsatisfied	Unsatisfied	Neutral	Satisfied	Very satisfied
Evacuation scenario	2 (2.6)	5 (6.5)	10 (13)	32 (41.6)	18 (23.4)
Daily tasks and functions	1 (1.3)	3 (3.9)	7 (9.1)	36 (46.8)	20 (26)
Memory test	1 (1.3)	0 (0)	12 (15.6)	35 (45.5)	19 (24.7)
Virtual museum	1 (1.3)	3 (3.9)	8 (10.4)	33 (42.9)	22 (28.6)
Virtual cafe	1 (1.3)	3 (3.9)	13 (16.9)	32 (41.6)	16 (20.8)

### Predicting the VR application usage likelihood

A standard multiple linear regression analysis was conducted to assess the predictors of older adults’ likelihood to use VR applications for dementia or cognitive impairment screening (Table 6). The regression model demonstrated statistical significance, *R* = .654, *R*² = 0.428, *F*(17, 59) = 2.60, *p* = .004. Education emerged as a positive predictor of the likelihood to use VR applications for screening (β = 0.269, *p* = .032), while the perceived utility of VR also positively predicted participants’ likelihood to use such applications (β = 0.334, *p* = .039). With a sample size of 77 participants, a post-hoc power analysis using G*Power calculated an observed power of 0.967. This high observed power indicates that our analysis has a 96.7% chance of detecting significant effects if they truly exist in the population, and suggests that our study is well-powered to identify meaningful relationships between the predictors and older adults’ likelihood to use VR applications for dementia screening.

**Table 6:** Coefficients and confidence intervals for multilinear regression

Variables		95% CI		
	B (*SE*)	LB	UB	β
Constant	4.785	− 0.858	10.427	
Age group	0.123	− 0.241	0.486	0.091
Gender	0.239	− 0.151	0.630	0.142
Country of birth	− 0.166	− 0.697	0.365	− 0.092
Socioeconomic status	0.058	− 0.088	0.205	0.116
Locality	− 0.064	− 0.590	0.461	− 0.028
***Education***	**0.360**	**0.031**	**0.690**	0.269*
Marital status	− 0.066	− 0.466	0.335	− 0.037
Employment status	0.121	− 0.682	0.925	0.041
Number of chronic conditions	− 0.043	− 0.232	0.145	− 0.057
Prior technology use	2.243	-3.870	8.357	0.765
Prior app use	-2.340	-8.456	3.777	− 0.804
Prior VR use	0.054	− 0.112	0.220	0.088
Perceived VR ease of use	− 0.297	− 0.746	0.151	− 0.326
***Perceived VR usefulness***	**0.321**	**0.017**	**0.624**	0.334*
Perceived ease of learning VR	− 0.177	− 0.479	0.125	− 0.222
Belief that technology can support health	− 0.090	− 0.291	0.111	− 0.108
Prior negative experiences with VR	− 0.536	-1.156	0.083	− 0.200

Note. Statistically significant results are bold and italicised

* *p* < .05

## Discussion

This study explored older adults’ acceptability of VR technology for dementia screening and the sociodemographic variables that predicted likelihood to adopt. Our results challenge conventional models that prioritise ease of use, instead emphasising the importance of educational levels and practical benefits in shaping the acceptance landscape of innovative healthcare technologies.

Consistent with prior research, our study indicates a general acceptance of VR applications among older adults, with positive perceptions regarding their usefulness [10, 18]. Participants predominantly favoured fully immersive and naturalistic VR applications, aligning with findings from Orr et al. [15] and highlighting the value of ecological validity in the design of VR screening applications to ensure alignment with user preferences [28]. The willingness of participants to share information derived from VR dementia screening applications with general practitioners also mirrors the trend observed with individuals using wearables for health information [21].

Furthermore, our finding that individuals with higher educational attainment, such as university graduates and postgraduates, exhibited a greater propensity to embrace VR technologies for dementia screening is consistent with existing literature [29–31]. This phenomenon aligns with the broader discourse on the impact of education on cognitive health, suggesting that individuals with more extensive educational backgrounds tend to exhibit enhanced cognitive reserve and adaptability [32–35]. One plausible interpretation of this educational divide in the acceptance of VR applications may be rooted in varying levels of technological literacy and exposure [36, 37]. Those with higher educational qualifications often possess a more extensive familiarity with technology, potentially rendering them more open to novel applications such as VR [38, 39]. Additionally, individuals with advanced educational backgrounds might have a greater appreciation for the potential benefits of technological advancements in healthcare, including early dementia screening through VR [40–42].

In contrast to the established Technology Acceptance Model (TAM) framework proposed by Davis [17], our findings highlight a divergence in technology adoption patterns among older adults, suggesting the need for a more balanced perspective. Unlike the conventional emphasis on ease of use within TAM, our research points towards the significance of perceived usefulness and the practical advantages of VR technologies in influencing the willingness of older individuals to adopt them. Considering alternative technology adoption models, such as the Unified Theory of Acceptance and Use of Technology (UTAUT) [43] or the Diffusion of Innovations theory [44], might provide additional insights into the multifactorial factors influencing the adoption of VR technologies among older adults. These models additionally consider factors such as social influence, facilitating conditions, and innovation characteristics, which could contribute to a more comprehensive understanding of early adopters compared to those in the late majority and influence the design and implementation of VR technologies in healthcare for older individuals.

Our study findings encourage a more comprehensive exploration of various theoretical perspectives and adoption frameworks. Further investigation into alternative factors and an in-depth exploration of elements contributing to long-term adoption and actual usage could enhance our understanding of the multifaceted factors influencing the adoption of VR technologies within digital healthcare use for older populations. This shift encourages a reconsideration of approaches within the healthcare domain, advocating for bespoke strategies that recognise and address the unique circumstances surrounding VR applications among older individuals, and particularly focused on sharing the benefits of technology use with this population to encourage its uptake.

### Strengths and limitations

Our findings present some of the first data available on sociodemographic, health status and technological acceptance predictors for older adults’ likelihood to adopt VR applications for dementia screening. In this respect, it provides preliminary evidence on the acceptability of VR applications for dementia screening among older adults. Second, this study also surveyed older adults’ preferences for VR technologies, which provides invaluable feedback for researchers, clinicians and policymakers who may decide to create novel VR applications for dementia screening. However, it should be noted that the sample size of this study was relatively small, which may increase the chance of falsely rejecting variables as unimportant and limiting the generalisability of the findings. A small sample size can further lead to reduced statistical power, making it more difficult to detect true effects or relationships within the data. This limitation should be considered when interpreting our study results, as it may affect the reliability and robustness of the conclusions drawn from the study. Future research with larger sample sizes would be beneficial to validate and extend the findings of this study.

Furthermore, our sample may not be fully representative of the broader population. Sociodemographic characteristics such as socioeconomic status and educational level varied within the sample, potentially introducing biases that could impact the generalizability of our results. Although efforts were made to recruit a diverse sample (33.8% of individuals were born in non-English speaking countries, and over 28% were from the lowest socioeconomic quintile, which is relatively representative of Australia’s national profile), these inherent variations may have influenced the outcomes observed in our study. Future studies should broaden their recruitment efforts to include a more diverse range of individuals. Additionally, it is important to acknowledge that our study used online recruitment methods, which may have influenced the results by potentially biasing towards individuals with internet access and technological literacy. Additional research could explore the contrast in attitudes obtained through in-person data collection, particularly among demographics without internet access.

Researchers should seek to collaborate with industry and healthcare partners to maximise access to ageing populations while benefiting from a diversity of inputs and potential co-design of novel VR applications for dementia screening. Finally, due to the emergent nature of VR applications in the ageing space, it may be possible that older adults are not yet fully informed about practicalities, benefits and disadvantages to using VR systems. Future research would benefit from including older adults in the design and development of new immersive VR applications prior to collecting acceptance data.

### Implications

Whilst acceptance of VR technology to screen for dementia was high in this current study, ethical considerations surrounding early screening for dementia and cognitive function in clinical practice should be contemplated when implementing such technologies. Although early detection of cognitive decline can enable timely interventions and support, it also raises concerns about overdiagnosis [45], labelling [46], and potential stigma associated with a dementia diagnosis [47]. Furthermore, implementing early screening measures may raise questions about patient autonomy, informed consent, and the potential impact of early diagnosis on older adults’ quality of life and mental wellbeing [48].

However, the benefits of early screening for dementia and cognitive impairment may potentially outweigh their limitations. In clinical practice, early diagnosis can inform personalised care plans, facilitate access to appropriate interventions and support services, and enable patients and their caregivers to make informed decisions about their health [49–51]. Healthcare professionals should also navigate ethical dilemmas such as balancing the benefits of early detection with the potential harms of false-positive results, as well as ensuring that screening processes are culturally sensitive, equitable, and respectful of individual preferences and values. Despite these considerations, recent studies indicate that both older adults [52] and healthcare professionals are receptive to using VR for screening for cognition [12]. Future research and practice should continue to prioritise the perspectives and voices of individuals with and without cognitive impairment in decision-making processes. This includes recognising that perceptions of cognitive impairment may evolve as symptoms progress, and adopting sensitive communication strategies tailored to individuals’ educational backgrounds and levels of cognitive impairment. Future research and clinical guidelines should continue to address these ethical considerations, ensuring that early screening practices are evidence-based, ethically sound, and aim to promote the overall wellbeing and autonomy of individuals.

## Conclusion

There is an increasing presence of digital health applications in healthcare, and emerging technologies can offer advantages over traditional approaches to dementia screening. Our findings contribute new insights into VR acceptability among older adults for dementia screening. Policymakers, clinicians, and researchers are encouraged to continue investing in VR applications for dementia screening, aligning with the imperative of understanding and catering to the needs of diverse populations to ensure the success of these healthcare innovations.

### Electronic supplementary material

Below is the link to the electronic supplementary material.

Supplementary Material 1

## Data Availability

Composite data can be made available upon reasonable request to the corresponding author (joyce.siette@westernsydney.edu.au).

## References

[1] World Health Organization. Decade of healthy ageing: baseline report. World Health Organization. 2021. https://www.who.int/publications/i/item/9789240017900. Accessed 18 February 2024.

[2] Langa KM. Cognitive aging, dementia, and the future of an aging population. In: Majmundar MK, Hayward MD, editors. Future directions for the demography of aging: Proceedings of a workshop; *2*018 Jun 26. Washington (DC). National Academies Press; 2018; pp. 249–268. https://www.ncbi.nlm.nih.gov/books/NBK513075/. 29989766

[3] GBD Dementia Forecasting Collaborators (2022). Estimation of the global prevalence of dementia in 2019 and forecasted prevalence in 2050: an analysis for the global burden of Disease Study 2019. Lancet Public Health.

[4] Appel L, Kisonas E, Appel E, Klein J, Bartlett D, Rosenberg J, Smith CN (2021). Administering virtual reality therapy to manage behavioral and psychological symptoms in patients with dementia admitted to an acute care hospital: results of a pilot study. JMIR Form Res.

[5] Dermody G, Whitehead L, Wilson G, Glass C (2020). The role of virtual reality in improving health outcomes for community-dwelling older adults: systematic review. J Med Internet Res.

[6] Javaid M, Haleem A (2020). Virtual reality applications toward medical field. Clin Epidemiol Glob Health.

[7] Jung T, tom Dieck MC, Lee H, Chung N. Effects of virtual reality and augmented reality on visitor experiences in museum. In: Inversini A, Schegg R, editors. Information and Communication Technologies in Tourism; 23 Jan 2016. Springer Cham; 2016. 10.1007/978-3-319-28231-2_45.

[8] Radianti J, Majchrzak TA, Fromm J, Wohlgenannt I (2020). A systematic review of immersive virtual reality applications for higher education: design elements, lessons learned, and research agenda. Comput Educ.

[9] Garrett B, Taverner T, Gromala D, Tao G, Cordingley E, Sun C (2018). Virtual reality clinical research: promises and challenges. JMIR Serious Games.

[10] Facal D, Spuch C, Valladares-Rodriguez S. New trends in cognitive aging and mild cognitive impairment. Geriatr (Basel). 2022;7(4). 10.3390/geriatrics7040080. 10.3390/geriatrics7040080 PMC9408095 36005256

[11] Siette J, Guion J, Ijaz K, Strutt P, Porte M, Savage G, Richards D. Development of a new computer simulated environment to screen cognition: assessing the feasibility and acceptability of Leaf Café in younger and older adults. BMC Med Inform Decis Mak. 202;424(1):79. 10.1186/s12911-024-02478-3. 10.1186/s12911-024-02478-3 PMC10949698 38504250

[12] Yondjo J, Siette J (2024). VR is the future: perspectives of healthcare professionals on virtual reality as a diagnostic tool for dementia status in primary care. BMC Med Inf Decis Mak.

[13] Kwon SH, Park JK, Koh YH (2023). A systematic review and meta-analysis on the effect of virtual reality-based rehabilitation for people with Parkinson’s disease. J Neuroeng Rehabil.

[14] Oliveira J, Gamito P, Souto T, Conde R, Ferreira M, Corotnean T, Fernandes A, Silva H, Neto T (2021). Virtual reality-based cognitive stimulation on people with mild to moderate dementia due to Alzheimer’s disease: a pilot randomized controlled trial. Int J Environ Res Public Health.

[15] Orr N, Yeo NL, Dean SG, White MP, Garside R. It makes you feel that you are there: exploring the acceptability of virtual reality nature environments for people with memory loss. Geriatr (Basel). 2021;6(1). 10.3390/geriatrics6010027. 10.3390/geriatrics6010027 PMC8005970 33809108

[16] Saredakis D, Keage HA, Corlis M, Loetscher T (2020). Using virtual reality to improve apathy in residential aged care: mixed methods study. J Med Internet Res.

[17] Davis FD. Perceived usefulness, perceived ease of use, and user acceptance of information technology. MIS Q. 1989;319–40. 10.2307/249008.

[18] Shu S, Woo BKP (2023). Pioneering the Metaverse: the role of the Metaverse in an aging population. JMIR Aging.

[19] Margrett JA, Ouverson KM, Gilbert SB, Phillips LA, Charness N (2022). Older adults’ use of extended reality: a systematic review. Front Virtual Real.

[20] Mondal P, Sinharoy A, Su L (2021). Sociodemographic predictors of COVID-19 vaccine acceptance: a nationwide US-based survey study. Public Health.

[21] Chandrasekaran R, Katthula V, Moustakas E (2021). Too old for technology? Use of wearable healthcare devices by older adults and their willingness to share health data with providers. Health Inf J.

[22] Schroeder T, Dodds L, Georgiou A, Gewald H, Siette J (2023). Older adults and new technology: mapping review of the factors associated with older adults’ intention to adopt digital technologies. JMIR Aging.

[23] Siette J, Seaman K, Dodds L, Ludlow K, Johnco C, Wuthrich V, Earl JK, Dawes P, Strutt P, Westbrook JI (2021). A national survey on COVID-19 second-wave lockdowns on older adults’ mental wellbeing, health-seeking behaviours and social outcomes across Australia. BMC Geriatr.

[24] Siette J, Dodds L, Brooks C, Deckers K (2023). Older adults’ perspectives towards optimizing lifestyle behaviors and strategies to support healthy brain ageing during COVID-19 restrictions. Front Public Health.

[25] Siette J, Dodds L, Deckers K, Köhler S, Armitage CJ (2023). Cross-sectional survey of attitudes and beliefs towards dementia risk reduction among Australian older adults. BMC Public Health.

[26] Tabachnick BG, Fidell LS (2019). Using multivariate statistics.

[27] Field A. Discovering statistics using IBM SPSS statistics. 4th ed. SAGE; 2013.

[28] Faria AL, Latorre J, Silva Cameirao M, Bermudez IBS, Llorens R (2023). Ecologically valid virtual reality-based technologies for assessment and rehabilitation of acquired brain injury: a systematic review. Front Psychol.

[29] Abd Majid F, Mohd Shamsudin N. Identifying factors affecting acceptance of virtual reality in classrooms based on technology acceptance model (TAM). Asian Journal of University Education. 2019;15(2):1–10. https://eric.ed.gov/?id=EJ1238733.

[30] Barrett AJ, Pack A, Quaid ED (2021). Understanding learners’ acceptance of high-immersion virtual reality systems: insights from confirmatory and exploratory PLS-SEM analyses. Comput Educ.

[31] Chau PH, Kwok YYJ, Chan MKM, Kwan KYD, Wong KL, Tang YH, Chau KLP, Lau SWM, Yiu YYY, Kwong MYF, Lai WTT, Leung MK (2021). Feasibility, acceptability, and efficacy of virtual reality training for older adults and people with disabilities: single-arm pre-post study. J Med Internet Res.

[32] Daffner KR (2010). Promoting successful cognitive aging: a comprehensive review. J Alzheimers Dis.

[33] Lovden M, Fratiglioni L, Glymour MM, Lindenberger U, Tucker-Drob EM (2020). Education and cognitive functioning across the life span. Psychol Sci Public Interest.

[34] Nelson ME, Veal BM, Andel R, Martinkova J, Veverova K, Horakova H, Nedelska Z, Laczó J, Vyhnalek M, Hort J (2022). Moderating effect of cognitive reserve on brain integrity and cognitive performance. Front Aging Neurosci.

[35] Phongpreecha T, Godrich D, Berson E, Espinosa C, Kim Y, Cholerton B, Chang AL, Mataraso S, Bukhari SA, Perna A, Yakabi K, Montine KS, Poston KL, Mormino E, White L, Beecham G, Aghaeepour N, Montine TJ (2023). Quantitative estimate of cognitive resilience and its medical and genetic associations. Alzheimers Res Ther.

[36] Baniasadi T, Ayyoubzadeh SM, Mohammadzadeh N (2020). Challenges and practical considerations in applying virtual reality in medical education and treatment. Oman Med J.

[37] Rong Q, Lian Q, Tang T (2022). Research on the influence of AI and VR technology for students’ concentration and creativity. Front Psychol.

[38] Bell IH, Nicholas J, Alvarez-Jimenez M, Thompson A, Valmaggia L (2020). Virtual reality as a clinical tool in mental health research and practice. Dialogues Clin Neurosci.

[39] Dwivedi YK, Hughes L, Baabdullah AM, Ribeiro-Navarrete S, Giannakis M, Al-Debei MM, Dennehy D, Metri B, Buhalis D, Cheung CM (2022). Metaverse beyond the hype: multidisciplinary perspectives on emerging challenges, opportunities, and agenda for research, practice and policy. Int J Inf Manage.

[40] Berridge C, Turner NR, Liu L, Karras SW, Chen A, Fredriksen-Goldsen K, Demiris G (2022). Advance planning for technology use in dementia care: development, design, and feasibility of a novel self-administered decision-making tool. JMIR Aging.

[41] Li R, Wang X, Lawler K, Garg S, Bai Q, Alty J (2022). Applications of artificial intelligence to aid early detection of dementia: a scoping review on current capabilities and future directions. J Biomed Inf.

[42] Muirhead K, Macaden L, Smyth K, Chandler C, Clarke C, Polson R, O’Malley C (2022). The characteristics of effective technology-enabled dementia education: a systematic review and mixed research synthesis. Syst Rev.

[43] Venkatesh V, Morris MG, Davis GB, Davis FD. User acceptance of information technology: toward a unified view. MIS Q. 2003;425–78. 10.2307/30036540.

[44] Rogers EM. Diffusion of innovations. 5th ed. Free; 2003.

[45] Maki Y (2021). Reconsidering the overdiagnosis of mild cognitive impairment for dementia prevention among adults aged ≥ 80 years. J Prim Health Care.

[46] Garand L, Lingler JH, Conner KO, Dew MA (2009). Diagnostic labels, stigma, and participation in research related to dementia and mild cognitive impairment. Res Gerontol Nurs.

[47] Siette J, Meka A, Antoniades J (2023). Breaking the barriers: overcoming dementia-related stigma in minority communities. Front Psychiatry.

[48] Whitehouse PJ (2019). Ethical issues in early diagnosis and prevention of Alzheimer disease. Dialogues Clin Neurosci.

[49] Milne A (2010). Dementia screening and early diagnosis: the case for and against. Health Risk Soc.

[50] Leifer BP (2003). Early diagnosis of Alzheimer’s disease: clinical and economic benefits. J Am Geriatr Soc.

[51] Robinson L, Tang E, Taylor JP (2015). Dementia: timely diagnosis and early intervention. BMJ.

[52] Siette J, Campbell C, Adam PJ, Harris CB (2024). Exploring the usability of the virtual reality module LEAF CAFÉ: a qualitative think-aloud study. BMC Geriatr.

