# Supplementary material for: Acceptability of virtual reality to screen for dementia in older adults
Source: BMC Geriatr. 2024 Jun 5;24:493. doi: 10.1186/s12877-024-05115-w (PMC11151481; doi:10.1186/s12877-024-05115-w)
Supplement: Supplementary file 1 — Supplementary Material 1 [file 12877_2024_5115_MOESM1_ESM.docx]

Supplementary Material

**Appendix A. Survey**

Start of Block: Part 1: Demographics

Q1 What is your age? ______

Q2 What is your gender?

- Female
- Male
- Non-binary/third gender
- Do not want to disclose

Q3  What is your place of birth?

- Australia
- UK
- New Zealand
- Italy
- China
- Lebanon
- Vietnam
- India
- Greece
- Other __________________________________________________

Q4 What is your cultural background?

- Lebanese
- Vietnamese
- Indian
- Chinese
- Greek
- Other __________________________________________________

Q5 What language do you speak at home?

- English
- Arabic
- Italian
- Indian
- Other __________________________________________________

Q6  What is your postcode?

________________________________________________________________

Q7 Which of the following best describes the highest educational qualification you have completed?

- Primary school (1)
- Secondary school (2)
- Trade qualification (eg carpentry) (3)
- Certificate (eg hairdressing, dress-making) (4)
- Diploma (5)
- Bachelor’s degree (6)
- Postgraduate Degree (7)

| Page Break |  |
| --- | --- |

Q8 What is your current marital status?

- Single (1)
- Married (2)
- De facto (3)
- Widowed (4)
- Divorced (5)
- Do not want to disclose (6)

Q9 What is your current employment status?

- Employed full-time (1)
- Employed part-time (2)
- Retired (3)
- Semi-retired (4)
- Full time home duties (5)
- Temporarily unable to work because of illness or injury (6)
- Permanently unable to work because of illness or injury (7)
- Unemployed (8)
- Student (9)

Q10 Have you had a diagnosis for any of the following?

- Chronic heart disease (1)
- Diabetes (2)
- Stroke (3)
- Sight impairment (4)
- Hearing impairment (5)
- Chronic obstructive respiratory disease (6)
- High blood pressure (7)
- Asthma (8)
- Dementia / Cognitive impairment (9)
- Depression / Anxiety (10)
- Other (12) __________________________________________________
- I do not have any chronic diseases/conditions (13)

End of Block: Part 1: Demographics

Start of Block: Part 2: E-health

Q1 How often do you need to have someone help you when you read instructions, pamphlets, or other written material from your doctor or pharmacy?

- Always
- Often
- Sometimes
- Occasionally
- Never

Q2 How confident are you filling out medical forms by yourself?

- Not at all
- A little bit
- Somewhat
- Quite a bit
- Extremely

Q3 Have you ever looked for information about health or medical topics from any online source?

- Yes
- No

Q4 Thinking about the most recent time you looked for information about health or medical topics, where or who did you go to first?

- Books (1)
- Brochures, pamphlets (4)
- Public Health Organization (5)
- Family (6)
- Friend/Co-worker (7)
- Doctor or healthcare provider (8)
- Internet (9)
- Library (10)
- Magazines (11)
- Newspapers (12)
- I have never looked for information about health or medical topics (13)

Q5 Please indicate how interested you are in using technology to help improve your health.

- Not interested at all
- Not interested
- Neutral
- Somewhat interested
- Very interested

Q6 During the past 6 months, have you used the Internet to look for health or medical information for yourself or others?

- Yes
- No

Q7 During the last 6 months, have you used any of the following to locate or share health information?

- Health applications
- Internet
- Social media (Facebook, Instagram)
- Blogs
- None of the above

Q8 How confident are you in using the information from the Internet to make health decisions?

- Not confident at all
- Not confident
- Neutral
- Confident
- Very confident

Q9 I know how to use the Internet to answer questions about my health.

- Yes
- Sometimes
- No

End of Block: Part 2: E-health

Start of Block: Part 3: Current experience with technology.

Q1 How often did you use the following technologies 
t**in the last 4 weeks?**

|  | A few times a day (12) | Once a day (13) | A few times a week (14) | Once a week (19) | Once a month (20) | Have never used (21) |
| --- | --- | --- | --- | --- | --- | --- |
| Phone (landline) (1) |  |  |  |  |  |  |
| Mobile phone (2) |  |  |  |  |  |  |
| Tablet or iPad (3) |  |  |  |  |  |  |
| Computer/laptop (4) |  |  |  |  |  |  |
| Wearable devices (e.g., Apple Watch, Garmin, Fitbit) (5) |  |  |  |  |  |  |
| Smart Home devices (e.g., Alexa, Google Home) (6) |  |  |  |  |  |  |

Q2 Please indicate how much you agree with this statement: "I am confident in using these devices generally."

- Strongly Agree (1)
- Agree (2)
- Neither agree nor disagree (3)
- Disagree (4)
- Strongly Disagree (5)

Q4 If you use the below apps, how often did you use them **in the last 4 weeks?**

|  | A few times a day (11) | Once a day (12) | A few times a week (13) | Once a week (14) | Once a month (15) | Have never used (16) |
| --- | --- | --- | --- | --- | --- | --- |
| Game based apps (e.g., Ward Search, angry Bird) (1) |  |  |  |  |  |  |
| Social media apps (e.g., Facebook, Twitter, WhatsApp, WeChat) (2) |  |  |  |  |  |  |
| Web browser (e.g., Safari, Internet Explorer, Chrome) (3) |  |  |  |  |  |  |
| Pre-installed apps (Camera, calculator, calendar) (4) |  |  |  |  |  |  |
| Video apps (Amazon, Disney, Netflix, YouTube) (5) |  |  |  |  |  |  |
| E-health application (e.g., Medicare, Pill Monitor, MedWatcher) (6) |  |  |  |  |  |  |

Q5 Virtual reality can be defined as a simulated environment in which an user experiences telepresence. Have you played or used any virtual reality games before?

- Yes
- No
- I don't know

Display This Question:

If Virtual reality can be defined as a simulated environment in which an user experiences telepresen... = Yes

Q6 Please specify what type of virtual reality you have played or used.

- Fully Immersive Virtual Reality (e.g., travelling inside the human body, feeling extreme close-up examination of objects)
- Non-immersive Virtual Reality (e.g., see the contents based on the device used, e.g., PC, smartphone, tablet
- Semi-Immersive Virtual Reality (e.g., a large screen, projection system and monitor - similar to IMAX cinemas)
- Augmented Reality (e.g., superimposing an image on your view of the real world, e.g., Google AR, L'Oreal's Midface)

Q6 What is your opinion of virtual reality technologies? Please indicate your agreement with the below statements

|  | Strongly disagree (18) | Disagree (19) | Neither Agree nor Disagree (20) | Agree (21) | Strongly Agree (22) | Not applicable (23) |
| --- | --- | --- | --- | --- | --- | --- |
| They are useful |  |  |  |  |  |  |
| They are fun |  |  |  |  |  |  |
| They are engaging |  |  |  |  |  |  |
| They are effective way in boosting my mood |  |  |  |  |  |  |
| They improve my social network |  |  |  |  |  |  |

End of Block: Part 3: Current experience with technology.

Start of Block:  Part 4: Ease of using virtual reality

Q1 Please indicate how much you agree with each of the below statements on your ease of using virtual reality.

|  | Strongly disagree (18) | Somewhat disagree (19) | Neither agree nor disagree (20) | Somewhat agree (21) | Strongly agree (22) |
| --- | --- | --- | --- | --- | --- |
| Learning to use virtual reality games would be easy for me (1) |  |  |  |  |  |
| My interaction with virtual reality is clear and understandable (2) |  |  |  |  |  |
| It would be easy for me to become skillful at using virtual reality (3) |  |  |  |  |  |
| I find virtual reality applications easy to use (4) |  |  |  |  |  |

| Page Break |  |
| --- | --- |

Q2 Here are some common challenges in using virtual reality technologies for older adults. Please rate how much you agree with these statements.

|  | Strongly disagree (6) | Somewhat disagree (7) | Neither agree nor disagree (8) | Somewhat agree (9) | Strongly agree (10) |
| --- | --- | --- | --- | --- | --- |
| They are expensive |  |  |  |  |  |
| They are not very accessible |  |  |  |  |  |
| They can make me feel sick |  |  |  |  |  |
| They aren’t many options available for me |  |  |  |  |  |

Q3 Have you experienced any of the following symptoms while using virtual reality? Select all that applies

- General discomfort
- Increased salivation
- Dizziness
- Vertigo
- Stomach awareness
- Burping
- Difficulties focusing
- Blurred vision
- None of the above

End of Block:  Part 4: Ease of using virtual reality

Start of Block: Part 5: Using virtual reality to screen for dementia

Q1 Virtual reality has been applied quite widely in the medical field. Please rate how much you agree with the below statements on how virtual reality technologies can be used to screen for dementia in older adults

|  | Strongly Disagree (6) | Disagree (7) | Neither Agree nor Disagree (8) | Agree (9) | Strongly Agree (10) |
| --- | --- | --- | --- | --- | --- |
| I think it’s a good idea |  |  |  |  |  |
| I think seniors will find it acceptable |  |  |  |  |  |
| I think seniors will find it useful |  |  |  |  |  |
| I would recommend the use for it |  |  |  |  |  |

End of Block: Part 5: Using virtual reality to screen for dementia

Start of Block: Part 6: Intention to use.

Q1 If a virtual reality application was developed to screen for dementia accurately, how likely are you to use it?

- Very likely
- Likely
- Neutral
- Unlikely
- Not at all likely

Q2 What format would you like to see the virtual application?

- Online website (7)
- Program to be downloaded on the computer (9)
- App to be downloaded on my phone or tablet (10)
- All of the above (11)

Q3 How immersive would you like the application to be?

- Fully immersive (1)
- Semi immersive (5)
- Non-immersive (6)
- I don’t know (7)

| Page Break |  |
| --- | --- |

Q4 Below are some applications of current virtual reality technologies used to screen for dementia. Please rate how much you would enjoy completing this application, if you had the opportunity.

|  | Very Unsatisfied (36) | Unsatisfied (37) | Neutral (38) | Satisfied (39) | Very Satisfied (40) |
| --- | --- | --- | --- | --- | --- |
| This is a naturalistic task that requires multitasking in a fire evacuation drill setting. There are 6 different simulated fire situations (from easy to more difficult) taking place at a virtual apartment block. You will be tested on your memory and reasoning in a emergency and evacuation routine. (5) |  |  |  |  |  |
| A 3D game that shows you a secret door which you have to open with the right key, and to make a phone call (by recalling a number). This game tests your everyday skills and memory. (6) |  |  |  |  |  |
| You are at a conference table with five adults and ready to be interviewed by these five avatars. You are asked to answer their questions as if they were real people. The avatars asked a series of questions, one at a time on a traditional memory test that you would get from a neuropsychologist. (7) |  |  |  |  |  |
| You visit a virtual museum and can choose an artifact to read. You are asked to remember as many details as you can from the archeological artifact within 5 minutes (color, shape, patterns, etc.) and will be tested on it later. (8) |  |  |  |  |  |
| Imagine you are working as a waiter/waitress at a cafe. You are meant to take orders for multiple customers, and are required to recall the orders later. The task gets harder, as more and more orders appear. (14) |  |  |  |  |  |

| Page Break |  |
| --- | --- |

Q5 How often would you want to use applications like the ones described earlier?

- Once a week (1)
- Once a month (4)
- Quarterly (5)
- Once every 6 months (6)
- Once a year (7)
- I don't want to use the ones above. Please tell us why. (8) __________________________________________________

Q6 Who would you like to have access to the information from the application?

- Family members (6)
- Friends (9)
- Partner (10)
- My GP (11)
- Other (12) __________________________________________________
- No one (13)

| Page Break |  |
| --- | --- |

Q8 And our final question - Do you have any other comments about the use of technology to screen for dementia?

________________________________________________________________

End of Block: Part 6: Intention to use.

Start of Block: Part 7:

Q40 Thank you very much for your time.

If you would like to enter the draw to win a $100 gift voucher please go to this link to enter your details: https://surveyswesternsydney.au1.qualtrics.com/jfe/form/SV_eEEkhnh7TarUbRQ

End of Block: Part 7:
